# Assessment of Electrospun Poly(ε-caprolactone) and Poly(lactic acid) Fiber Scaffolds to Generate 3D In Vitro Models of Colorectal Adenocarcinoma: A Preliminary Study †

**DOI:** 10.3390/ijms24119443

**Published:** 2023-05-29

**Authors:** Claudio Ricci, Bahareh Azimi, Luca Panariello, Benedetta Antognoli, Beatrice Cecchini, Roberta Rovelli, Meruyert Rustembek, Patrizia Cinelli, Mario Milazzo, Serena Danti, Andrea Lazzeri

**Affiliations:** 1 Department of Civil and Industrial Engineering, University of Pisa, Largo L. Lazzarino 2, 56126 Pisa, Italy; 2 Centre for Instrumentation Sharing of University of Pisa (CISUP), Lungarno Pacinotti 43/44, 56126 Pisa, Italy

**Keywords:** Caco-2 cells, mechanical properties, metabolic activity, intestine model

## Abstract

Three-dimensional scaffold-based culture has been increasingly gaining influence in oncology as a therapeutic strategy for tumors with a high relapse percentage. This study aims to evaluate electrospun poly(ε-caprolactone) (PCL) and poly(lactic acid) (PLA) scaffolds to create a 3D model of colorectal adenocarcinoma. Specifically, the physico-mechanical and morphological properties of PCL and PLA electrospun fiber meshes collected at different drum velocities, i.e., 500 rpm, 1000 rpm and 2500 rpm, were assessed. Fiber size, mesh porosity, pore size distribution, water contact angle and tensile mechanical properties were investigated. Caco-2 cells were cultured on the produced PCL and PLA scaffolds for 7 days, demonstrating good cell viability and metabolic activity in all the scaffolds. A cross-analysis of the cell–scaffold interactions with morphological, mechanical and surface characterizations of the different electrospun fiber meshes was carried out, showing an opposite trend of cell metabolic activity in PLA and PCL scaffolds regardless of the fiber alignment, which increased in PLA and decreased in PCL. The best samples for Caco-2 cell culture were PCL500 (randomly oriented fibers) and PLA2500 (aligned fibers). Caco-2 cells had the highest metabolic activity in these scaffolds, with Young’s moduli in the range of 8.6–21.9 MPa. PCL500 showed Young’s modulus and strain at break close to those of the large intestine. Advancements in 3D in vitro models of colorectal adenocarcinoma could move forward the development of therapies for this cancer.

## 1. Introduction

The choice of the appropriate material and production technique for a scaffold to be used in specific anatomic environments is essential to ensure a positive outcome in terms of biocompatibility, cell differentiation, mechanical match with surrounding tissues and degradation time [1]. The scaffold material can be of natural origin, such as chitosan and collagen, or produced synthetically, such as poly(ε-caprolactone) (PCL), poly(glycolic acid) (PGA) and poly(lactic acid) (PLA). These aliphatic polyesters are endowed with hydrolytic biodegradation and general biocompatibility with many tissues and organs [2]. Most of all, the use of these polymers is advantageous in terms of material reproducibility and good processability [3,4,5,6]. Among the different techniques used to fabricate scaffolds, electrospinning has emerged as one of the most convenient and efficient [7,8]. In fact, this technique allows three-dimensional (3D) fibrous meshes to be fabricated, with a high surface area, interconnected pores and mechanical and structural stability. An electrospun structure favors the organization of cells, provides signals for cellular responses and supports cell adhesion, proliferation and migration, while allowing cell differentiation [9]. Since PCL and PLA are widely used polymers in the biomedical field, over the years, scientists have managed to optimize the production parameters to obtain tissue-specific electrospun scaffolds based on these polymers [10,11,12,13,14]. Several different solutions of PCL and PLA have been employed to produce electrospun scaffolds to obtain morphological, mechanical and chemical properties suitable for diverse body tissues [15,16,17,18,19].

An interesting application of electrospun scaffolds concerns the development of tissue-engineered tumor models [19,20,21]. The development of in vitro 3D cancer models using different types of scaffolds represents a novel tissue engineering approach, which has been acquiring considerable relevance in recent years. Biomaterial-based scaffolds provide cells with a 3D supporting structure that ultimately acts as a preliminary extracellular matrix (ECM), enabling mechanical, chemical and topographical features that can accomplish the characteristics of a specific tissue, where the tumor originates. Culturing cancer cells within scaffolds could provide useful tools to better understand and study the tumor microenvironment and, therefore, could be used to investigate tumor-specific treatments for patients [22,23,24]. 

To obtain homogeneous, fibrous meshes for tissue engineering using electrospinning technology, researchers have proved that PCL–chloroform represents an appropriate polymer–solvent system [25,26]. For example, Lowery et al. produced randomly oriented PCL fibers by electrospinning a 10 *w*/*v*% PCL/chloroform solution, which were successfully employed to culture human dermal fibroblasts [27]. Concerning cancer models, in a recent study, Permlid et al. demonstrated the possibility of successfully developing an in vitro breast cancer model culturing two different malignant breast cancer lines on a highly porous PCL-based 3D scaffold produced via electrospinning [25]. Their results showed that all cells could penetrate the fibrous scaffold producing a 3D structure that resembled the tumor. A successful application of PLA-based scaffolds was proposed by Polonio-Alcalà et al., who produced an in vitro 3D breast cancer model based on an electrospun PLA scaffold [21]. They cultured a triple-negative breast cancer cell line on the scaffold, obtaining a model able to mimic the physiological conditions of the tumor in vivo and the high cell proliferation rates. In the form of bulk materials, PCL and PLA show different mechanical properties, which can be exploited to target the diverse stiffness in pathologic tissues [28]. It has been demonstrated that many cancerous cells are very sensitive to substrate stiffness [29,30]. Among these, a colorectal tumor has recently been pointed out to be regulated according to matrix stiffness via the progression, proliferation, invasion and metastasis of colorectal cancer cells across a number of pathways [31]. Colorectal cancer originates from the rectum and represents a major cause of cancer-related mortality in the Western world. In vitro 3D cancer models could contribute to better understanding this tumor and its correlation with extracellular matrix (ECM) mechanics.

This study aimed to produce and compare the electrospun fiber scaffolds of PCL and PLA as potential substrates for colorectal cancer cells. The fibrous meshes were obtained using a rotating collector at increasing velocity to induce fiber alignment (i.e., 500 rpm, 1000 rpm and 2500 rpm), and characterized from a physico-mechanical standpoint, including fiber morphology, size, mesh porosity, hydrophilicity and tensile mechanical properties. Finally, these scaffolds were cultured in vitro with Caco-2 cells; therefore, cell viability, metabolic activity and morphology were investigated. A novel 3D in vitro model to study colorectal cancer by virtue of the scaffold properties can open new avenues for understanding biological mechanisms in view of targeted therapies. 

## 2. Results

### 2.1. Morphological Characterization

**Figure 1:**
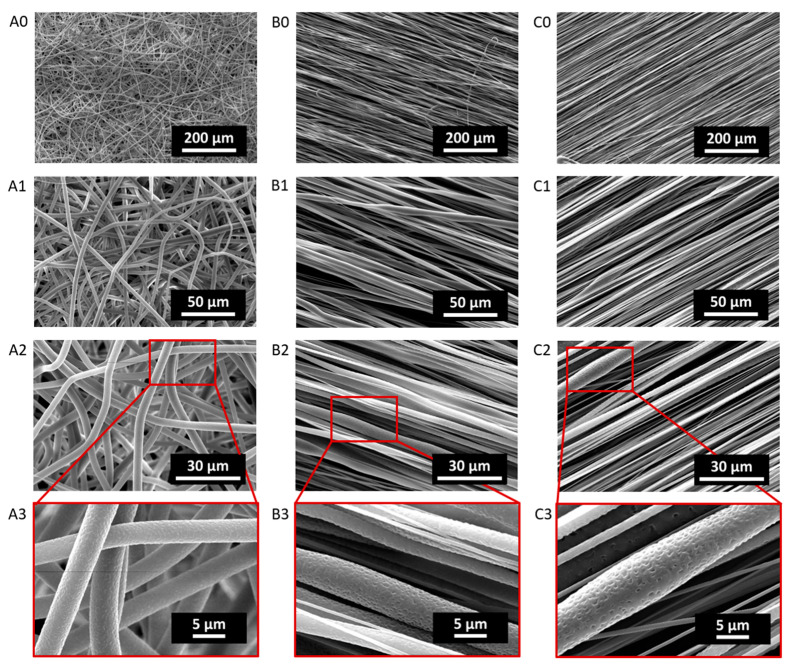
SEM micrographs of PCL500 (**A0**–**A3**), PCL1000 (**B0**–**B3**) and PCL2500 (**C0**–**C3**), acquired at 500× (**A0**,**B0**,**C0**), 2000× (**A1**,**B1**,**C1**) and 4000× (**A2**,**B2**,**C2**) magnifications and 15 kV. Lens showing fiber surface nanoporosity (**A3**,**B3**,**C3**).

**Figure 2:**
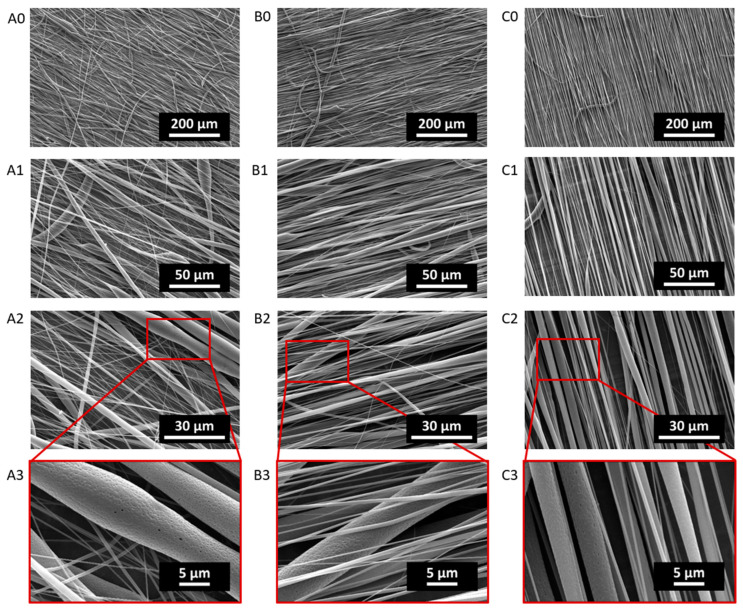
SEM micrographs of PLA500 (**A0**–**A3**), PLA1000 (**B0**–**B3**) and PLA2500 (**C0**–**C3**) acquired at 500× (**A0**,**B0**,**C0**), 2000× (**A1**,**B1**,**C1**) and 4000× (**A2**,**B2**,**C2**) magnifications and 15 kV. Lens showing fiber surface nanoporosity (**A3**,**B3**,**C3**).

Micrographs of electrospun PCL and PLA fiber scaffolds collected at 500 rpm, 1000 rpm and 2500 rpm are reported in Figure 1 and Figure 2, respectively, showing increasing magnifications as obtained via scanning electron microscopy (SEM) analysis. SEM micrographs were used to evaluate the fiber diameter, as reported in Table 1.

The PCL500 resulted in a randomly oriented fibrous mesh, whose fibers were characterized by a constant uniform diameter of 3.10 ± 0.12 µm across the fiber length (Figure 1A1–A3). While PCL fibers collected at higher speeds (i.e., PCL1000 and PCL2500) were increasingly more aligned, their fiber diameters resulted in being less homogeneous. In fact, some of the thicker fibers showed a non-constant diameter across the fiber length, as observed in Figure 1(B1–B3,C1–C3) for the PCL1000 and PCL2500, respectively. Moreover, as the alignment of the fibers increased, the average fiber diameter decreased. In particular, PCL1000 fiber diameter was 1.83 ± 0.45 µm, while PCL2500 fiber diameter was 0.95 ± 0.49 µm.

Overall, both PCL and PLA fibers displayed a decrease in the average fiber diameter with an increasing rotation speed of the collector (i.e., alignment). However, it has to be noted that the magnification provided by the SEM images was not sufficient to measure the diameter of the thinnest fibers with great accuracy. Moreover, higher-magnification images (2000×, 4000×) revealed the presence of tiny pores on the surface of the fibers, for both PCL and PLA. Details of the surface porosity of the fibers can be observed for the PCL and PLA fibers in Figure 1A3–C3 and Figure 2A3–C3, respectively. 

### 2.2. Porosity and Pore Size Distribution 

The numerical values of the porosity, in terms of average values and standard deviation, of the fabricated scaffolds are summarized in Table 2. The porosity of both the PCL and PLA scaffolds decreased with increasing fiber alignment. Therefore, among the PCL scaffolds, the PCL500 achieved the highest porosity value of 87.57 ± 4.74%, while the PCL1000 and PCL2500 showed a porosity of 81.77 ± 2.06% and 79.54 ± 1.98%, respectively. Concerning the PLA scaffolds, the PLA500 showed a porosity of 79.80 ± 2.18%, while the PLA1000 and PLA2500 showed a porosity of 74.45 ± 3.02% and 64.20 ± 1.24%, respectively. Therefore, the PCL scaffolds resulted in being more porous than those made of PLA. Statistically, significant differences for both materials were detected when the collector velocity increased. Similarly, there were significant differences between groups belonging to different polymers at equal collector speeds (detailed results are reported in Table S1 in the Supplementary Material).

The pore diameters of the PCL and PLA electrospun fiber scaffolds are reported in Table 3. The PCL fibrous scaffolds showed larger pores than the PLA counterparts. Therefore, PLC2500 reached the highest pore diameter value of 8.56 ± 2.97 µm, while PCL1000 and PCL500 showed average pore diameters of 7.00 ± 5.00 µm and 8.05 ± 5.11 µm, respectively. Concerning the PLA scaffolds, the pore diameters increased with increasing fiber alignment. Thus, PLA500 resulted in having the lowest pore size of 3.52 ± 1.40 µm, while PLA1000 and PLA2500 exhibited pore sizes of 4.61 ± 2.20 µm and 6.50 ± 4.6 µm, respectively. From a statistical standpoint, significant differences between material groups at similar collector speeds were detected. In contrast, within samples of the same polymer, significant differences at different collector speeds were noticeable only for the PLA fiber meshes (detailed results are reported in Table S2 in the Supplementary Material).

**Figure 3:**
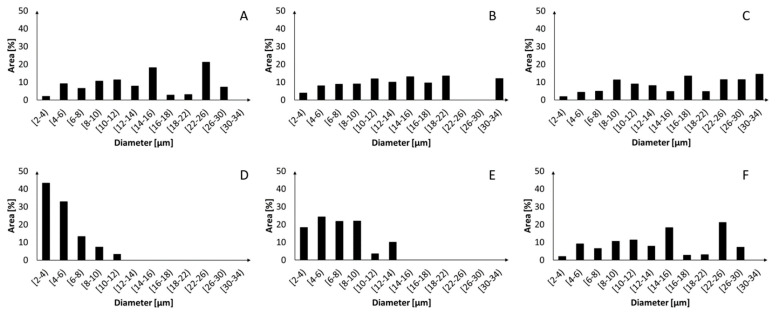
Pore diameter distribution for PCL500 (**A**), PCL1000 (**B**), PCL2500 (**C**), PLA500 (**D**), PLA1000 (**E**) and PLA2500 (**F**) electrospun meshes. The pore sizes of both the PCL and PLA scaffolds increased as the rotation speed of the collector increased. The PCL fiber meshes showed larger pores than the PLA counterparts collected at the same speed. This difference was less relevant at high collection speeds.

The pore size distribution graphs of the PCL and PLA scaffolds are reported in Figure 3. Both the PCL and PLA scaffolds showed an increased dimension of the pores with increasing fiber alignment. Specifically, the largest pores of the PCL500 samples had equivalent diameters in the 26–30 µm range, while the largest pores of the PCL1000 and PCL2500 samples showed equivalent diameters in the 30–34 µm range. On the other hand, the PLA500 samples displayed pores whose largest equivalent diameter was in the 10–12 µm range, whereas the PLA1000 and PLA2500 showed their largest pores in the 12–14 µm and 22–26 µm ranges, respectively. Overall, the PCL scaffolds were characterized by larger pores with respect to the corresponding PLA scaffolds. However, this difference became less pronounced as the fiber alignment increased. In summary, with increasing fiber alignment, the mesh became denser (i.e., less porous), even though larger inter-fiber pores (>30 µm equivalent diameter for PCL and >15 µm equivalent for PLA) were present.

### 2.3. Mechanical Characterization 

**Figure 4:**
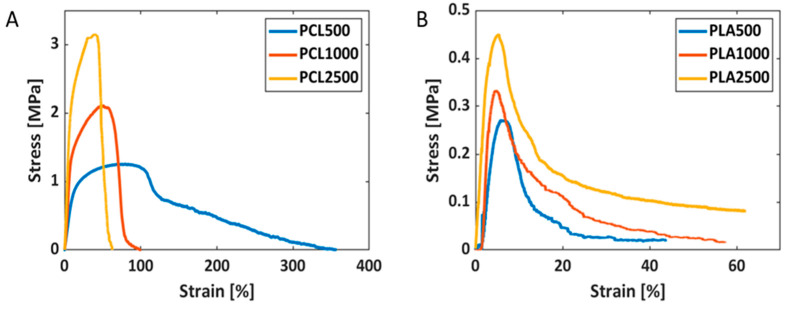
Tensile test curves of PCL (**A**) and PLA (**B**) fiber meshes electrospun at 500 rpm, 1000 rpm and 2500 rpm. The fibers showed increasing strength and stiffness as the collection speed increased.

The stress–strain curves of the PCL and PLA electrospun scaffolds, performed at 5 mm/min, are reported in Figure 4. 

All the results related to the mechanical characterization, including tensile strength, strain at break, Young’s modulus and toughness, are given in Table 4, while the outcomes of the statistical analyses are reported in Table S3 (Supplementary Materials).

Among the PCL scaffolds, a trend was observed in the mechanical properties. On average, the PCL500 samples showed the lowest Young’s modulus (8.35 MPa) and tensile strength (1.70 MPa), whereas the PCL2500 samples showed the highest Young’s modulus (37.25 MPa) and tensile strength (3.29 MPa). Consistently, these mechanical properties of PCL1000 fell in between the ones of the PCL500 and PCL2500 samples. On average, the PCL500 fiber meshes demonstrated the highest strain at break with a value of 71.83%, whereas the PCL1000 and PCL2500 fiber meshes showed strains at break of 47.00% and 38.72%, respectively. Therefore, as the fiber alignment increased, Young’s modulus and the tensile strength increased, while the strain at break decreased. The PCL500 samples’ average toughness was 0.74 MJ/m^3^; in contrast, the PCL1000 and PCL2500 samples showed higher values of 0.77 MJ/m^3^ and 1.65 MJ/m^3^, respectively. 

The PLA scaffolds showed considerably poorer mechanical properties with respect to the PCL counterparts. Specifically, the tensile strength of all of the scaffolds was in the order of hundreds of kPa. The PLA500 samples revealed the lowest tensile strength and Young’s modulus, whose average values were equal to 0.24 MPa and 4.56 MPa, respectively. The PLA1000 and PLA2500 samples showed average tensile strengths of 0.35 MPa and 0.50 MPa, respectively, and average Young’s moduli of 14.69 MPa and 15.38 MPa, respectively. Different from the PCL fiber meshes, in the PLA samples, the strain at break increased with an increasing rotational speed of the collector (i.e., fiber alignment degree). The PLA500 fiber meshes showed a mean strain at break of 4.56%, while the PLA1000 and PLA2500 fiber meshes showed 4.91% and 5.13%, respectively. Therefore, a general trend could be observed for the strain at break and Young’s modulus, even though the value detected in PLA1000 was on average slightly higher than that of PLA2500, but this was not statistically different.

It was possible to observe a trend in the values of toughness. An increase in toughness with increasing fiber alignment was obtained, even though it was more than an order of magnitude lower than the PCL counterparts. In fact, the PLA500 samples showed toughness of 0.03 MJ/m^3^, PLA1000 achieved 0.04 MJ/m^3^ and PLA2500 showed 0.11 MJ/m^3^. 

### 2.4. Water Contact Angle

All the scaffolds presented water contact angle (WCA) values greater than 90°, as a sign of their hydrophobicity. Moreover, for both the PCL and PLA scaffolds, it was possible to observe an increase in the water contact angle value (i.e., hydrophobicity) with increasing fiber alignment. The average WCAs were 101.5° (PCL500), 103.7° (PCL1000) and 106.0° (PCL2500). The PLA samples were slightly more hydrophobic than the PCL samples, showing average WCAs of 110.0° (PLA500), 119.3° (PLA1000), and 121.8° (PLA2500). 

The WCA results are summarized in Table 5.

### 2.5. Cell Culture

**Figure 5:**
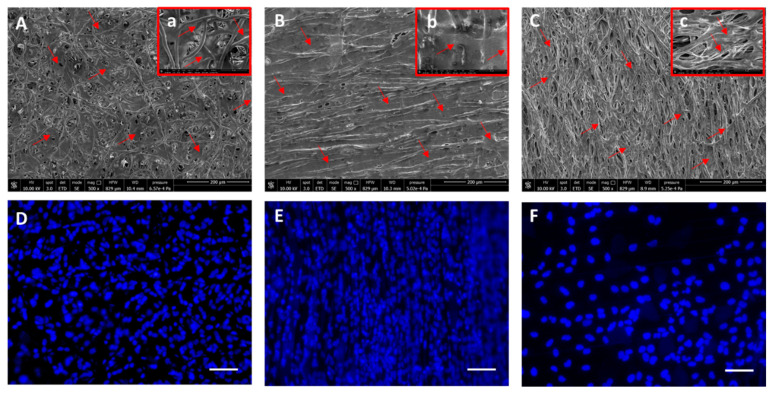
SEM and fluorescence microscopy analysis of electrospun PCL fibers seeded with Caco-2 cells. (**A**–**C**): SEM analysis; (**a**–**c**): zoomed in magnifications of each image showing cell colonization of the fibers. (**D**–**F**): DAPI (cell nuclei in blue); (**A**,**D**): 500 rpm; (**B**,**E**): 1000 rpm; (**C**,**F**): 2500 rpm fiber collection speeds. Red arrows: Caco-2 cells; scale bar = 100 µm. Lenses (**a**–**c**) show details of cells within the fiber pores.

**Figure 6:**
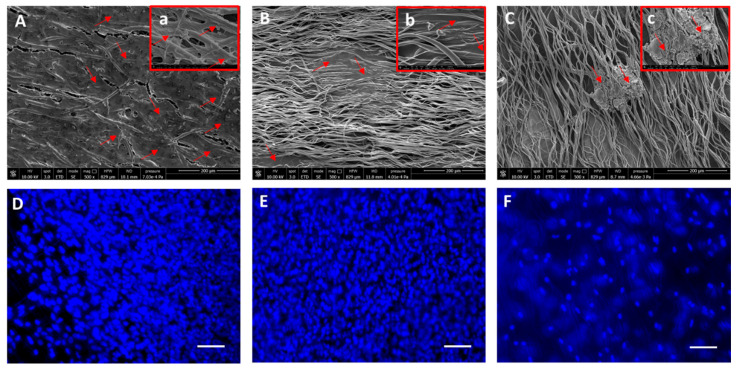
SEM and fluorescence microscopy analysis of PLA electrospun fibers seeded with Caco-2 cells. (**A**–**C**): SEM analysis; (**a**–**c**): zoomed in magnifications of each image showing cell colonization of the fibers. (**D**–**F**): DAPI (cell nuclei in blue); (**A**,**D**): 500 rpm; (**B**,**E**): 1000 rpm; (**C**,**F**): 2500 rpm fiber collection speeds. Red arrows: Caco-2 cells; scale bar = 100 µm. Lenses (**a**–**c**) show details of cells within the fiber pores.

The PCL and PLA fiber scaffolds were cultured in vitro for 7 days using Caco-2 cells. The results of morphological analysis conducted using SEM and DAPI staining are shown in Figure 5 and Figure 6 for PCL and PLA fiber meshes, respectively. The obtained findings demonstrated that both the PCL and PLA scaffolds were widely colonized by Caco-2 cells on the top surface and sometimes within the fibers. Many nuclei were imaged in all of the scaffold types. The random fiber scaffolds (i.e., collected at 500 rpm) showed high cell density on the scaffold surface (Figure 5A,D and Figure 6A,D).

**Figure 7:**
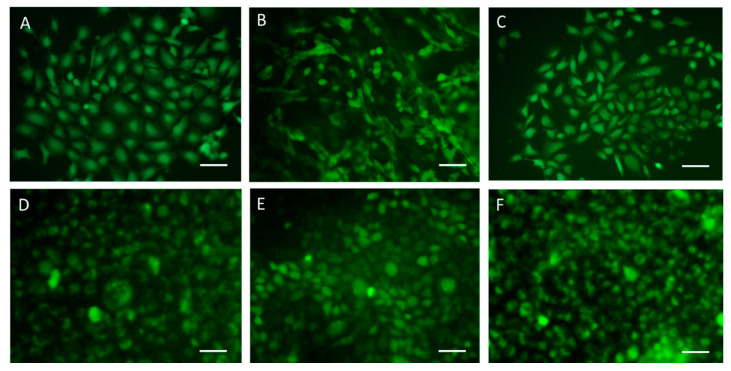
Fluorescence Live/Dead analysis of Caco-2 cells cultured on (**A**–**C**) PCL and (**D**–**F**) PLA electrospun fibers collected at (**A**,**D**) 500 rpm, (**B**,**E**) 1000 rpm and (**C**,**F**) 2500 rpm drum velocities**.** Micrographs were acquired using 100× original magnification; scale bar = 100 µm. Live cells were stained with Calcein AM and are shown in green.

In the PCL1000 (Figure 5B,E) and PLA1000 (Figure 6B,E) fiber scaffolds, dense cell layers were visible via SEM and fluorescence microscopy on the top surface of the scaffolds, which followed the orientation of the fibers. The PCL and PLA fibers collected at 2500 rpm showed less cells on the top surface, along with the presence of cells at different depth levels (Figure 5C,F and Figure 6C,F). The cells were viable in all of the scaffolds, as detected using the Live/Dead assay via fluorescence microscopy and reported in Figure 7.

**Figure 8:**
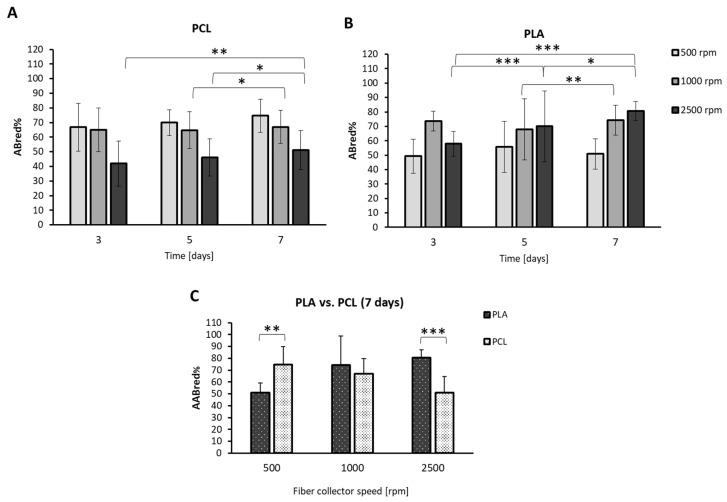
Bar graphs showing the results of metabolic activity, evaluated as resazurin reduction percentage (AB_red_%) with respect to negative control using Alamar Blue assay, and of Caco-2 cells cultured on (**A**) PCL and (**B**) PLA electrospun fiber scaffolds, collected at 500 rpm, 1000 rpm and 2500 rpm, during 7-day cultures (*n* = 3). (**C**) Comparison of the obtained results at the endpoint (i.e., PLA versus PCL). Data are expressed as mean ± standard deviation. Statistical analysis was performed via ANOVA; * *p* < 0.05, ** *p* < 0.001 and *** *p* < 0.0001.

The results of the Alamar Blue test conducted every 2 days for 1 week are shown in Figure 8. The Alamar Blue assay measured the metabolic activity of the cells in culture. This test was performed using the modality against negative controls (i.e., only scaffolds + dye, without cells) to observe the increase in metabolic activity over time and compare it among 3D samples.

The randomly oriented fibers (i.e., collected at 500 rpm) for both PCL and PLA demonstrated steady-state metabolic activity of the Caco-2 cells over time, which was higher overall in the PCL samples (*p* = n.s.) (Figure 8A,B). In PCL1000, a statistically significant increase (*p* < 0.05) in metabolic activity was detected from Day 5 to Day 7 (Figure 8A). Similarly, in PLA1000, a statistically significant increase (*p* < 0.001) was observed in the same timeframe (Figure 8B). 

In the samples collected at 2500 rpm, both in PCL and in PLA, the metabolic activity increased between all of the timepoints with statistical significance, even if the average values detected in the PCL scaffolds were always lower than those of the PLA counterparts (Figure 8A,B). Finally, by comparing the obtained findings for each group (PLA versus PCL) at the endpoint (i.e., 7 days in culture), as reported in Figure 8C, in the randomly oriented fiber configuration, the metabolic activity of the Caco-2 cells was higher, with statistical significance (*p* < 0.001), in PCL500 than in PLA500, whereas, in the most aligned fiber configuration, it was higher in the PLA2500 (*p* < 0.0001) than in the PCL2500 scaffolds. In contrast, the cell metabolic activity in the PCL1000 and PLA1000 scaffolds resulted in being not statistically different (*p* = n.s.). These outcomes demonstrate that not only the fiber alignment, but also other properties of the scaffolds, concur with the cellular response.

## 3. Discussion

It has recently been pointed out that 3D in vitro cancer tissue models can reproduce cancer features very similar to those present in native tumors and can be viable for many weeks, making it possible to study cancer biology and assess therapeutic efficacy in a more reliable way than conventional cell cultures [30]. This study investigates PLA and PCL scaffolds to support Caco-2 cell culture, with the aim of enabling 3D in vitro cancer models useful for a future new generation of dedicated targeted therapies. In addition, it provides a comprehensive physico-mechanical characterization of fibrous polymer scaffolds. Two different polymers, namely PCL and PLA, were used to prepare electrospun fibrous meshes at different collection speeds of the rotating drum. Both of these biomaterials have been widely employed as scaffolds, and in general proved to be suitable for the growth of many cell types [16,17]. Even though both PCL and PLA belong to the aliphatic polyester family, show predominant hydrolytic biodegradation in the human body and are hydrophobic in nature, they possess different mechanical properties, which may affect the biological response. For example, PLA is known to be brittle, whereas PCL is a more ductile polymer than PLA; therefore, PLA/PCL blends were largely investigated to accomplish the desired mechanical properties [31,32]. Under the tissue engineering paradigm, the scaffold acts as a preliminary ECM. It has been unveiled that ECM stiffness can play a key role in tumorigenesis, including in colorectal cancer [31]. Therefore, PCL and PLA scaffolds may induce different biological behavior with colorectal cancer cells, due to their specific physico-mechanical properties. Improving the insight into cancer cell behavior could result in the development of better biomimetic 3D cancer models and the consequent assessment of more effective therapies than the ones currently available.

In this study, we produced PCL and PLA fiber meshes using electrospinning with an identical solvent system and polymer concentration. The fibers were collected under increasing velocity of the rotative drum, which gave rise to fiber structures spanning from randomly oriented (at 500 rpm) to uniaxially aligned (at 2500 rpm). An extensive characterization of the scaffolds was performed, including fiber mesh morphology, fiber size, mesh thickness and porosity, mesh pore diameter distribution, wettability and a panel of mechanical properties (i.e., tensile strength, strain at break, Young’s modulus and toughness of the meshes). In fact, not only the mechanical properties, but also other physical properties, which can change with the fiber alignment degree, have been demonstrated to concur with cell response [33,34]. Thereafter, all the produced scaffolds were cultured with Caco-2 cells, as a cellular model of colorectal adenocarcinoma. 

Starting from a morphological analysis, the homogeneity of the fiber size in the PCL scaffolds can be ascribed to the correct choice of polymer–solvent system. In fact, as has been widely reported in the literature, chloroform results in being a good solvent for PCL in the electrospinning process, leading to the obtainment of homogeneous-sized ultrafine fibers. Moreover, the lack of beaded structures in any of these scaffolds suggests that 12.5 w/v% was a suitable PCL concentration in chloroform [27,35]. Chloroform was chosen as a solvent phase for both PCL and PLA since the Hildebrand solubility parameter (δ) of chloroform (9.3 cal^1/2^∙cm^3/2^) is close to that of PCL (10.0 cal^1/2^∙cm^3/2^) and PLA (10.0 cal^1/2^∙cm^3/2^) [11,36]; thus, we expected to also observe homogeneous fiber formation in PLA meshes. However, the SEM analysis of the PLA fiber meshes revealed some inhomogeneities, which could be ascribed to a not-ideal concentration of PLA in chloroform, or to the possibility of PLA–chloroform not being an ideal system for electrospinning applications, as suggested by other authors who have experienced similar issues [10,13]. Regardless of the different morphologies, all of the produced scaffolds had distinct fibers, whose diameters fell in the expected range according to the literature [24,37].

Other important characteristics for a scaffold, crucial for a successful cell culture, are pore size and porosity. To this end, theories have been developed to assess the relationships between pore size, fiber diameter and porosity in electrospun fiber networks [38]. PCL scaffolds present larger pores with respect to the corresponding PLA ones. This result agrees with the porosity associated with the scaffolds. In fact, PCL scaffolds showed a higher percentage of porosity than the PLA ones. The increase in the broadness of the pore size distribution with the increasing collector speed was related to the morphological properties of the meshes. In general, highly aligned fibers show a lower porosity than their random fiber counterparts, but the pores are expected to be larger as they are obtained via considering the interstices between almost parallel fibers [7]. On the other hand, in randomly oriented fibers, the porosity is generally higher than that in aligned fibers, due to the loose fibers. Moreover, pore size is expected to be smaller as the pores are formed at the interstices among fibers whose orientation is completely random. 

Under our processing and environmental conditions, i.e., 41% relative humidity and using chloroform as a solvent, surface nanoporous fibers were formed in all of the PLA and PCL scaffolds. Since PCL and PLA are hydrophobic polymers, at high relative humidity, the surface of the fibers is wetted, and water molecules coalesce into water droplets that ultimately leave nanosized indentations on the fiber surface [7]. Surface nanoporosity could be a desirable feature, as the surface area is increased, and a non-smooth topography could enhance cell adhesion to the substrate.

The mechanical properties of a scaffold are crucial in terms of cell–scaffold interactions. Depending on the cell type, an ideal scaffold stiffness is able to induce specific cellular mechanisms [39]. We observed that the PCL and PLA scaffolds increased their mechanical properties upon fiber alignment. The PCL500 and PLA500 fiber meshes thus showed the lowest Young’s moduli and tensile strengths, due to the mobility of their non-oriented fiber networks. In contrast, the PCL2500 and the PLA1000 fiber meshes presented the highest Young’s moduli and tensile strengths. This trend has been confirmed by previous studies on polymeric fiber meshes [23,24,25], and can be due to the different morphologies characterizing the scaffolds [24,40,41]. In fact, as the fiber alignment increases, the scaffold becomes more anisotropic, showing higher mechanical properties in the direction of the orientation of the fibers. Since tensile tests were performed in the direction of the fibers, the results obtained confirmed the expectations. As all of the stretched fibers contribute to the tensile force, uniaxially aligned fibers usually show the highest tensile modulus. Moreover, PCL scaffolds showed a decrease in the strain at break with an increase in the fiber alignment, which was expected because of their increased anisotropy. However, this trend was not observed for the PLA scaffolds, possibly due to the low thickness of the samples obtained, which made their testing more difficult. Anyway, all of the PLA scaffolds showed stress–strain behavior typical of the electrospun PLA fibers [42]. 

Concerning scaffold wettability, which is known to affect cell adhesion [43,44], the WCA values measured for the different scaffolds were in agreement with those reported in the literature [45,46,47]. Since both PCL and PLA are hydrophobic polymers, combinations with hydrophilic molecules or post-treatments are used to modify the surface properties of the scaffolds [48]. Results for both polymers showed an increase in scaffold hydrophobicity, with increasing fiber alignment, which can be related to the reduction in scaffold porosity with increasing fiber alignment [49]. In fact, water molecules in a scaffold with lower porosity cannot easily penetrate the insides of the mesh, which results in the formation of a more compact droplet. Moreover, the PLA scaffolds resulted in being more hydrophobic than the PCL scaffolds. This behavior can be further explained by considering the porosity results. In fact, the PLA scaffolds resulted in being less porous than the PCL counterparts. Therefore, this property can contribute to increase the hydrophobicity of PLA scaffolds.

Caco-2 is an immortalized cell line of human colorectal adenocarcinoma cells, with heterogeneous morphology, also used to mimic the normal intestinal barrier. In this study, we used Caco-2 cells in combination with the produced electrospun scaffolds to screen the best candidates to focus on the generation of 3D colorectal models. As such, the biological investigation was performed for a short culture time (i.e., 7 days). The necessity to progress in colorectal adenocarcinoma 3D models has been underpinned by several authors, who used bioprinting, alginate bead biofabrication, hydrogels, organoids and microfluidic devices [50,51,52,53]. Electrospun PLA nanofibrous scaffolds have recently been used to generate a model of the intestinal barrier, with Caco-2 cells cultured for 4 days [54]. 

Our study aimed to investigate the best PCL and PLA electrospun scaffolds to culture Caco-2 cells for generating a 3D adenocarcinoma in vitro model. In our preliminary study, it was observed that all of the produced scaffolds supported the adhesion and viability of Caco-2 cells for 1 week, as demonstrated via SEM and fluorescence microscopy analyses, as well as cell metabolic activity detection, with the latter being a dominant sign in cancer development and progression [55]. However, some interesting differences were observed. Although no relevant diversity could be found between the PLA1000 and PCL1000 scaffolds according to cell colonization and metabolic activity, as both showed good performance, in contrast, PCL500 and PLA2500 induced significantly higher metabolic activity in the Caco-2 cells with respect to the PLA500 and PCL2500 counterparts. Overall, PCL500–1000 and PLA1000–2500 showed good cellular outcomes with Caco-2 cells. These scaffolds covered a wide set of different physical, morphological and mechanical properties, which were difficult to correlate. Thus, we also searched for any morphological and physical–mechanical factors possibly responsible for the opposite metabolic activity trend observed in the PCL500–2500 and PLA500–2500 scaffolds detected at 7 days of culture with Caco-2 cells. In our study, the only property that showed an opposite trend in the PCL and PLA series was elongation at break, though with one-order-of-magnitude-different values between the two.

It is interesting to note that the PCL500 scaffolds had the lowest WCA and the highest porosity, and vice versa for the PLA2500 scaffolds. A peculiar difference between the PCL500 and PLA500 scaffolds was the pore size distribution that highlighted the presence of large pores (reaching 15–30 µm) only in PCL500, whereas Young’s moduli were similarly low in both scaffold types. The highest metabolic activity was achieved in the samples with Young’s moduli ranging from about 8 to 23 MPa, namely, those of the PCL500–1000 and PLA1000–2500 scaffolds. There is not a defined range for this scaffold property in relation to colorectal cancer. The mechanical properties of a normal large intestine have been extensively studied. An average value of 5.18 ± 0.24 MPa for Young’s modulus has been reported, as well as an average value of 62.8% ± 0.4% for strain at break, measured in the tensile mode [56]. These values are very close to those averagely observed for PCL500, i.e., 8.35 MPa for Young’s modulus and 71.83% strain at break, thus suggesting that PCL500 can be used as a suitable scaffold for the colon–rectum. This is consistent with the results of metabolic activity, and excludes PLA500, which showed a Young modulus still comparable to that of the intestine, i.e., 7.61 MPa, but an average strain at break of only 4.56%.

A different approach evaluates the mechanical properties of the colorectal tumor tissue in the compressive mode or via ultrasound elastography, which is useful for diagnostic purposes. There are not many studies that try to correlate the scaffold properties with the colorectal cancer cell behavior. Using polyacrylamide gels and HCT-8 colorectal cancer cells, researchers determined a range of 21–47 kPa for Young’s modulus [57]. 

Overall, PCL and PLA electrospun fibers showed an opposite trend, giving increased metabolic activity as a function of fiber alignment in PLA, and vice versa in PCL fiber meshes. Further investigations must be performed to assess whether other tumor features, such as oncogene expression, will show a difference in these models. Understanding the complexity of the tumor microenvironment would enable the possibility of designing cancer-specific 3D in vitro models, personalized for the patient [58]. 

## 4. Materials and Methods

### 4.1. Materials

PCL with a number average molecular weight (Mn) of 80,000 g/mol and a melting temperature (T_m_) of 60°C (code: 440744-20G) was supplied by Sigma-Aldrich (Milan, Italy) in pellet form. PLA (Ingeo Biopolymer 2003D) with a density of 1.24 g/cm^3^ and a melting temperature (T_m_) of 151°C was purchased from NatureWorks^®^ (Minneapolis, MN, USA) in pellet form. Chloroform (EMPROVE^®^ ESSENTIAL) (CAS-No: 67-66-3) was supplied by Merck KGaA (Darmstadt, Germany). Caco-2 cells were obtained from ATCC, LGC Standards GmbH (Wesel, Germany). RPMI culture medium, phosphate-buffered saline (PBS), penicillin-streptomycin, L-glutamine and trypsin were purchased from Merk KGaA. Alamar Blue dye, 4′,6-diamidino-2-phenylindole (DAPI) and a Live/Dead cell imaging kit were obtained from Thermo Fisher Scientific (Waltham, MA, USA). 

### 4.2. Electrospinning Process

PCL and PLA solutions for electrospinning were prepared by dissolving the polymer in chloroform at a concentration of 12.5 *w*/*v*% [11,36]. The solutions were magnetically stirred at room temperature (RT) and maintained at the stirring speed of 300 rpm overnight, until uniform appearance.

Electrospinning was performed using an electrospinning bench apparatus (Linari Engineering s.r.l., Pisa, Italy) to fabricate fibrous meshes of PCL and PLA. Each freshly prepared solution was loaded into a 5 mL glass syringe, fitted with a blunt-tip stainless steel needle and then continuously fed at a constant flow rate of 1.0 mL/h via a syringe pump (NE-300; New Era Pump Systems, Inc., Farmingdale, NY, USA). Electrospinning was conducted at 15 kV using a high-voltage power supply (S1600079 Linari High Voltage; Linari Engineering s.r.l.) consisting of a ground terminal attached to the metal needle and a positive terminal connected to a rotating collector. The 8 cm diameter drum collector (Linari Engineering s.r.l., Pisa, Italy) was covered with aluminum foil and placed at a needle tip distance of 10 cm for PCL and 15 cm for PLA solutions. The production of the electrospun fiber meshes was performed by collecting the polymeric fibers onto the rotating drum using 3 different collection speeds: 500 rpm, 1000 rpm and 2500 rpm, for both the PCL and PLA polymer solutions. For each sample, the electrospinning time was fixed at 30 min and all of the fabrication steps were performed at RT with 41% relative humidity. 

### 4.3. Morphological Characterization 

The morphology of the scaffolds was investigated via field emission scanning electron microscopy (FE-SEM) using a FEI FEG-Quanta 450 instrument (Field Electron and Ion Company, Hillsboro, OR, USA). The acquired images were analyzed using Image J Software (version 1.53). The fibers’ average diameter was calculated from at least 100 measures for both the PCL and PLA scaffolds and the results were reported along the associated standard deviation. 

### 4.4. Porosity and Pore Size Distribution

The porosity percentage value of each scaffold was calculated using ImageJ Software (version 1.53) on 9 micrographs. An appropriate threshold was set for each 500× magnification SEM image of the scaffolds, to only consider the first layer of fibers. The pore size distribution was derived from the measurements of the pore areas (at least 100 pores per sample) obtained applying ImageJ tools on the 2000× magnification SEM images. The equivalent pore diameter was calculated for each pore and the results obtained were reported in graphs as area (%) vs. diameter for each scaffold.

### 4.5. Mechanical Characterization 

The tensile strength, Young’s modulus and the strain at break of the scaffolds were determined using an Instron 5500R universal testing machine equipped with a 100 N load cell and pneumatic grips, interfaced with MERLIN software (version 4.42), used in the tensile mode. To perform the test, the electrospun scaffolds were cut in the direction of the fibers, as suggested by a previous study available in the literature [59], to obtain test specimens of a length of 20 mm and a width of 10 mm. Prior to the tensile test, the thickness of each specimen was measured in 3 different areas using a feeler gauge (Mitutoyo Italiana s.r.l., Lainate, Italy) and an average was taken. To perform the tensile test, 3 × 1 cm^2^ specimens were secured in a paper cage with dimensions of 3 × 3 cm^2^ to avoid the direct contact between the clamps and the fibers, and the test was run at a constant velocity of 5 mm/min after having set the initial grip distance at 20 mm. The stress was calculated by considering the ratio between the force and the initial geometrical cross-sectional area of the specimen (thickness × width), while the tensile modulus was calculated from the slope of the stress–strain curve in the linear region. The maximum value of stress was considered as the break point for each scaffold. The area under the stress–strain curve was calculated to obtain a measurement of the scaffold toughness. 

### 4.6. Water Contact Angle

The static water contact angle was measured at RT using a contact angle goniometer (Ramé-Hart 100-00 230 NRL). Five droplets of 3 µL of distilled water were dropped from a single-use plastic syringe on the surface of the rectangular 5 × 1 cm^2^ scaffold sample placed on the goniometer stage. For each scaffold, only one sample was obtained and tested, while for each sample, the water contact angle was measured for at least two droplets on both sides and the average value of the readings was reported, along with the associated standard deviation.

### 4.7. Culture of Caco-2/Scaffold Constructs

We used Caco-2 cells for the in vitro studies. The scaffolds were sterilized overnight via UV irradiation. The cells were cultured in 75 cm^2^ tissue culture flasks using RPMI medium supplemented with 1% penicillin-streptomycin, 1% L-glutamine and 10% fetal calf serum in a humidified incubator set at 37 °C with 95% air and 5% CO_2_. When the 80% confluence was reached, the cells were detached via enzymatic action using trypsin, and their number was determined using a Burker chamber. Caco-2 cells were seeded with a density of 2.5 × 10^5^ cells/mm^3^ (scaffold volume) in 30 µL of culture medium. The samples were thus incubated for 1 h to ensure cell adhesion. Afterwards, the specimens were completely covered by the entire RMPI medium and cultured for 7 days, and the medium was changed every 3 days.

### 4.8. Cell Morphology and Viability

A viability test was performed using a Live/Dead assay at the endpoint. The live samples were added with sterile PBS, containing the fluorescent dyes according to the manufacturer’s protocol, and were observed under a fluorescence inverted microscope equipped with a camera (Nikon-Ti, Tokyo, Japan). At the endpoint, the samples were fixed in 10% formalin for 10 min at 4 °C, rinsed in distilled water and dried in a vacuumed oven set at 37 °C. The specimens were mounted on aluminum stumps, sputter-coated with platinum and observed via FE-SEM, as described in Section 4.3. A set of samples were incubated in 10 µg/mL DAPI solution in the dark to detect cell nuclei and were observed under fluorescence microscopy, as reported above.

### 4.9. Alamar Blue Assay

The Alamar Blue assay was used to test the viability of the cell/scaffold constructs. The dye was incubated for 3 h with the samples and controls, including scaffolds with no cells as negative controls, according to the manufacturer’s instructions. To determine the cell metabolic activity, samples were tested at different culture time periods (3, 5 and 7 days). The supernatants were taken from the cultures after each experiment and replaced with fresh culture media. A spectrophotometer (Victor3, PerkinElmer, Waltham, MA, USA) was used to evaluate the samples using a double-wavelength reading of 570 nm and 600 nm. Finally, the dye reduction (%) was computed using the dye molar extinction values and appropriate absorbance formulae, as provided by the manufacturer [60].

### 4.10. Statistical Analysis

Statistical analysis was carried out to discuss the significance of the differences observed between the scaffold groups. Independent *t*-student test analyses were performed using Jamovi Software (V. 2.2.5), taking account of the numerosity of the samples and setting a significance probability threshold (*p*) equal to 0.05.

## 5. Conclusions

Developing 3D in vitro models of colorectal adenocarcinoma is expected to move forward the discovery of better effective therapies for this cancer. We studied PCL and PLA fiber scaffolds obtained via electrospinning polymeric solutions in chloroform under three different velocities of the rotative collector, i.e., 500 rpm, 1000 rpm and 2500 rpm. By changing the polymer and the fiber alignment, hydrophobic properties, pore size and porosity changed, along with mechanical properties. Caco-2 cells were able to colonize all of the produced scaffolds. However, an opposite trend of cell metabolic activity occurred in the PLA and PCL scaffolds as a function of the fiber alignment, which increased in PLA and decreased in PCL scaffolds. The highest metabolic activity outcomes were found in PCL500 (randomly oriented fibers) and PLA2500 (aligned fibers). The suitable scaffolds, with high metabolic activity, including PCL1000 and PLA1000, showed Young’s moduli in the range of 8–23 MPa. Among all of them, the PCL500 scaffolds had the most similar Young moduli and strain at break to those of the large intestine. In conclusion, many parameters resulted in affecting the Caco-2 cell behavior on the PLA and PCL fiber scaffolds, thus unveiling more efforts to be performed along the pathway for understanding cancer development and progression. 

## Figures and Tables

**Table 1:** Scaffold thickness and fiber diameter of PCL and PLA scaffolds electrospun at 500 rpm, 1000 rpm and 2500 rpm; fiber diameters are reported as mean value ± standard deviation. The fibers showed increasingly smaller diameters when the rotation speed of the collector increased.

Polymer	Collector Speed (rpm)	Mesh Thickness (µm)	Fiber Diameter (μm)
PCL	500	50 ± 5	3.10 ± 0.12
	1000	45 ± 6	1.83 ± 0.45
	2500	40 ± 5	0.95 ± 0.49
PLA	500	33 ± 7	2.59 ± 2.35
	1000	103 ± 6	2.07 ± 2.16
	2500	113 ± 8	1.76 ± 1.50

**Table 2:** Porosity of electrospun PCL and PLA fiber scaffolds collected at 500 rpm, 1000 rpm, and 2500 rpm. Both PCL and PLA samples showed a decrease in the porosity when the rotation speed of the collector increased. PCL fiber meshes were more porous than the PLA counterparts electrospun at the same speed.

Sample	Porosity (%)
PCL500	87.57 ± 4.74
PCL1000	81.77 ± 2.06
PCL2500	79.54 ± 1.98
PLA500	79.80 ± 2.18
PLA1000	74.45 ± 3.02
PLA2500	64.20 ± 1.24

**Table 3:** Pore diameters of electrospun PCL and PLA fiber scaffolds collected at 500 rpm, 1000 rpm and 2500 rpm. PCL scaffolds presented larger pore diameters with respect to the PLA counterparts.

Sample	Pore Diameter (µm)
PCL500	8.05 ± 5.11
PCL1000	7.00 ± 5.00
PCL2500	8.56 ± 2.97
PLA500	3.52 ± 1.40
PLA1000	4.61 ± 2.20
PLA2500	6.50 ± 4.60

**Table 4:** Tensile strength, Young’s modulus, strain at break and toughness of PCL and PLA fiber scaffolds electrospun using 500 rpm, 1000 rpm and 2500 rpm collector speeds. The samples showed increasing strength and toughness as the collection speed increased.

Sample	Tensile Strength (MPa)	Strain at Break (%)	Young’s Modulus (MPa)	Toughness (MJ/m^3^)
PCL500	1.70 ± 0.46	71.83 ± 7.32	8.35 ± 0.27	0.74 ±0.14
PCL1000	1.97 ± 0.54	47.00 ± 3.60	22.28 ± 1.07	0.77 ± 0.45
PCL2500	3.29 ± 0.19	38.72 ± 7.18	37.25 ± 5.95	1.65 ± 0.55
PLA500	0.24 ± 0.09	4.56 ± 1.14	7.61 ± 0.37	0.03 ± 0.01
PLA1000	0.35 ± 0.02	4.91 ± 0.69	14.69 ± 0.88	0.04 ± 0.01
PLA2500	0.50 ± 0.06	5.13 ± 0.04	15.38 ± 1.93	0.11 ± 0.01

**Table 5:** Water contact angle (WCA) measurements of PCL and PLA fiber scaffolds electrospun using 500 rpm, 1000 rpm and 2500 rpm collector velocities. The WCAs are reported as mean value ± standard deviation. The WCAs of both the PCL and PLA samples increased with increasing collection speed, showing progressively higher hydrophobic behavior. The PCL samples exhibited lower WCAs than those of the respective PLA counterparts.

Scaffold Type	WCA (°)
PCL500	101.5 ± 3.5
PCL1000	103.7 ± 0.6
PCL2500	106.0 ± 1.4
PLA500	110.0 ± 0.5
PLA1000	119.3 ± 1.1
PLA2500	121.8 ± 0.4

## Data Availability

Data are available upon request to the corresponding author.

## Supplementary Materials

The supporting information can be downloaded at https://www.mdpi.com/article/10.3390/ijms24119443/s1.
