# Supplementary material for: Assessment of Electrospun Poly(ε-caprolactone) and Poly(lactic acid) Fiber Scaffolds to Generate 3D In Vitro Models of Colorectal Adenocarcinoma: A Preliminary Study
Source: Int J Mol Sci. 2023 May 29;24(11):9443. doi: 10.3390/ijms24119443 (PMC10253282; doi:10.3390/ijms24119443)
Supplement: Supplementary file 1 [file ijms-24-09443-s001.zip › ijms-2400160-supplementary.pdf]

Table S1. Statistical analyses concerning the porosity of the scaffolds.

PCL	500 rpm	1000 rpm
**1000 rpm**	0.066	-
**2500 rpm**	< 0.001	0.003

PLA	500 rpm	1000 rpm
**1000 rpm**	< 0.001	-
**2500 rpm**	< 0.001	< 0.001

PCL vs. PLA	500 rpm	1000 rpm	2500 rpm
	0.002	< 0.001	< 0.001

Table S2. Statistical analyses concerning the pore sizes of the scaffolds.

PCL	500 rpm	1000 rpm
**1000 rpm**	0.11	-
**2500 rpm**	0.49	0.06

PLA	500 rpm	1000 rpm
**1000 rpm**	< 0.001	-
**2500 rpm**	< 0.001	< 0.001

PCL vs. PLA	500 rpm	1000 rpm	2500 rpm
	< 0.001	< 0.001	0.003

Table S3. Statistical analyses concerning the mechanical properties of the scaffolds.

Statistical significance for the Tensile Strengths

PCL	500 rpm	1000 rpm
**1000 rpm**	0.539	-
**2500 rpm**	0.005	0.016

PLA	500 rpm	1000 rpm
**1000 rpm**	0.122	-
**2500 rpm**	0.015	0.014

PCL vs. PLA	500 rpm	1000 rpm	2500 rpm
	0.006	0.006	< 0.001

Statistical significance for the Strain at break

PCL	500 rpm	1000 rpm
**1000 rpm**	0.006	-
**2500 rpm**	0.001	0.149

PLA	500 rpm	1000 rpm
**1000 rpm**	0.668	-
**2500 rpm**	0.432	0.612

PCL vs. PLA	500 rpm	1000 rpm	2500 rpm
	< 0.001	< 0.001	0.001

Statistical significance for the Young's Modulus

PCL	500 rpm	1000 rpm
**1000 rpm**	< 0.001	-
**2500 rpm**	0.005	0.013

PLA	500 rpm	1000 rpm
**1000 rpm**	< 0.001	-
**2500 rpm**	0.002	0.112

PCL vs. PLA	500 rpm	1000 rpm	2500 rpm
	0.046	0.018	0.004

Statistical significance for the Toughness

PCL	500 rpm	1000 rpm
**1000 rpm**	0.924	-
**2500 rpm**	0.051	0.096

PLA	500 rpm	1000 rpm
**1000 rpm**	0.147	-
**2500 rpm**	< 0.001	0.002

PCL vs. PLA	500 rpm	1000 rpm	2500 rpm
	< 0.001	0.048	0.009
